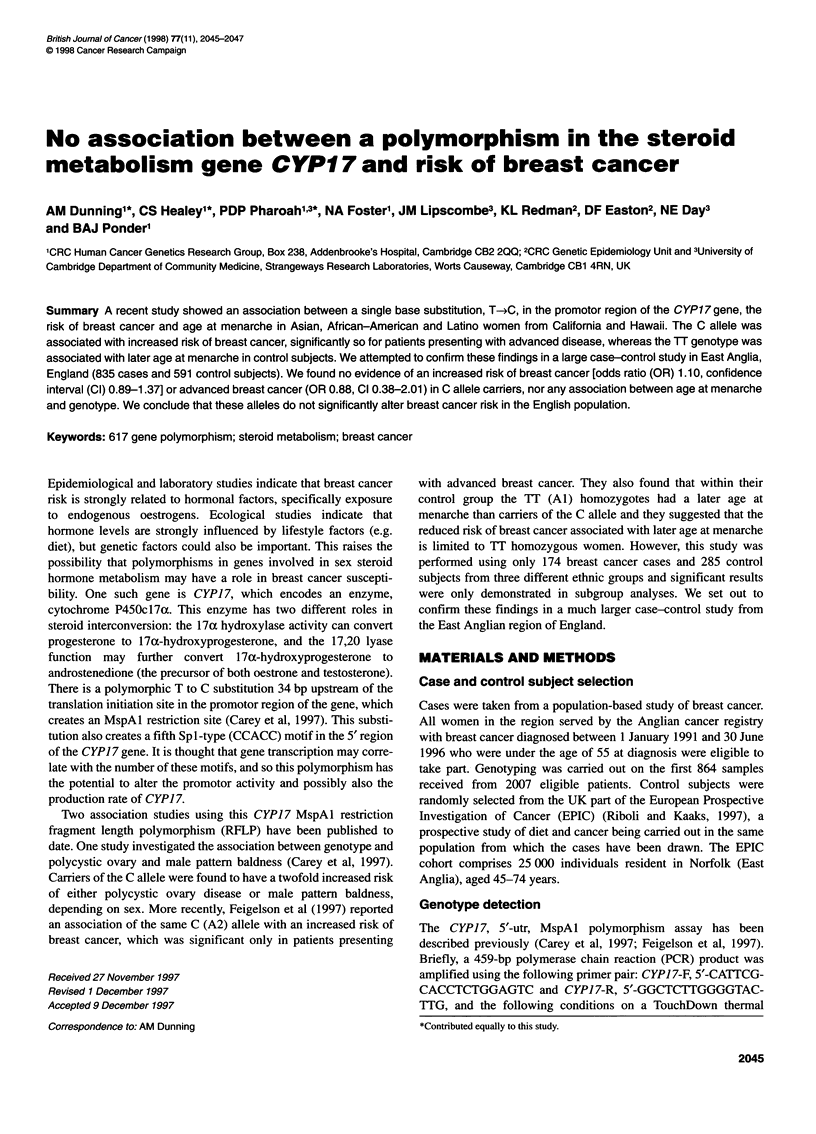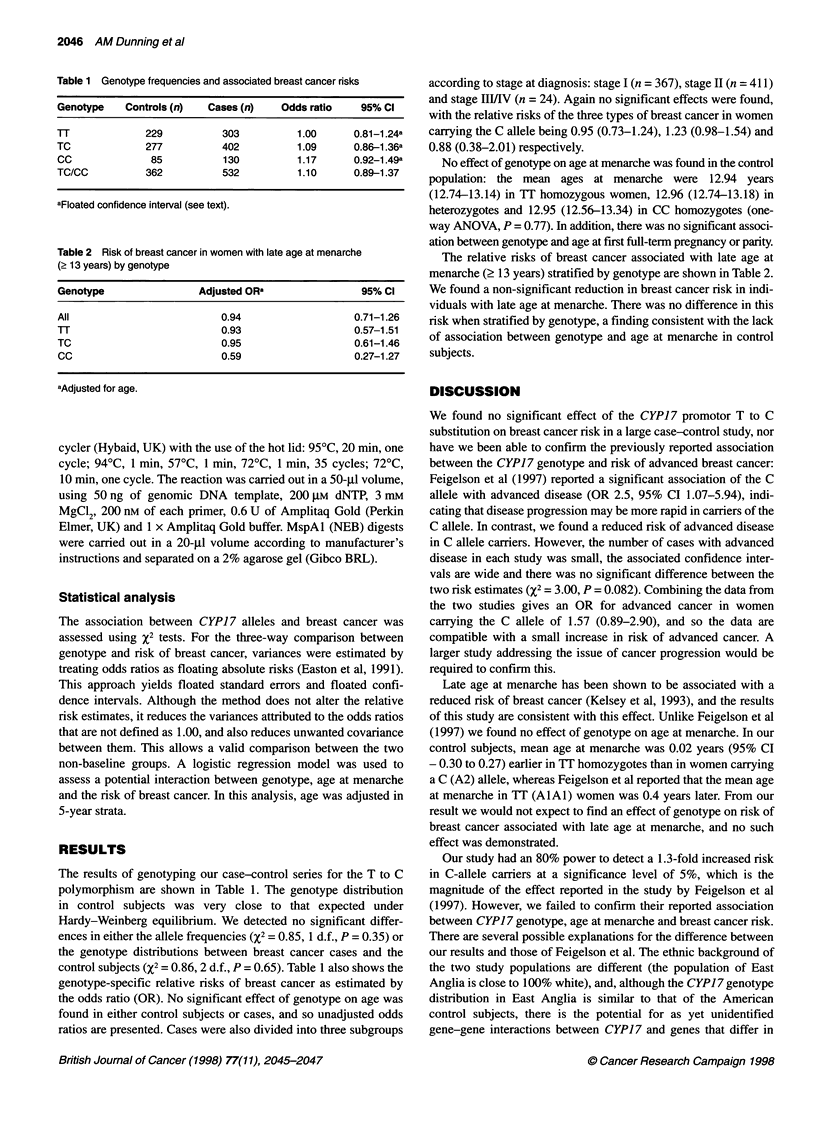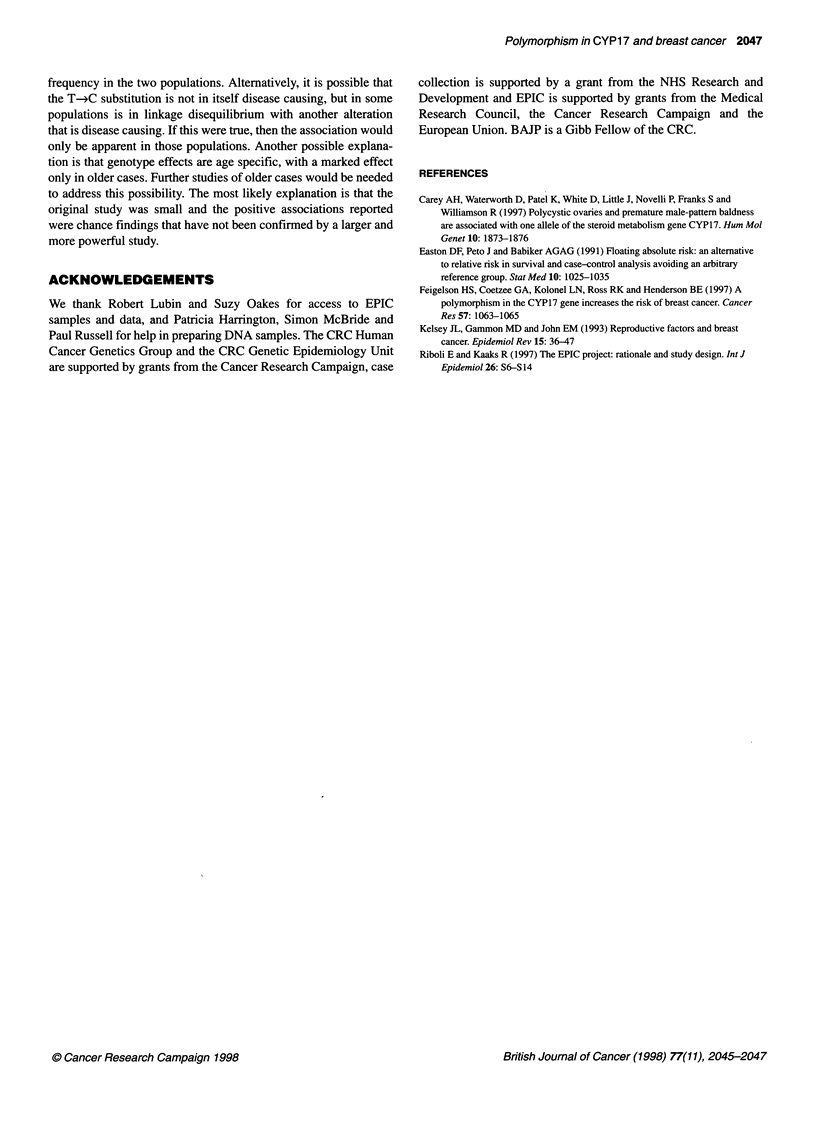# No association between a polymorphism in the steroid metabolism gene CYP17 and risk of breast cancer.

**DOI:** 10.1038/bjc.1998.340

**Published:** 1998-06

**Authors:** A. M. Dunning, C. S. Healey, P. D. Pharoah, N. A. Foster, J. M. Lipscombe, K. L. Redman, D. F. Easton, N. E. Day, B. A. Ponder

**Affiliations:** CRC Human Cancer Genetics Research Group, Addenbrooke's Hospital, Cambridge, UK.

## Abstract

A recent study showed an association between a single base substitution, T-->C, in the promotor region of the CYP17 gene, the risk of breast cancer and age at menarche in Asian, African-American and Latino women from California and Hawaii. The C allele was associated with increased risk of breast cancer, significantly so for patients presenting with advanced disease, whereas the TT genotype was associated with later age at menarche in control subjects. We attempted to confirm these findings in a large case-control study in East Anglia, England (835 cases and 591 control subjects). We found no evidence of an increased risk of breast cancer [odds ratio (OR) 1.10, confidence interval (CI) 0.89-1.37] or advanced breast cancer (OR 0.88, CI 0.38-2.01) in C allele carriers, nor any association between age at menarche and genotype. We conclude that these alleles do not significantly alter breast cancer risk in the English population.


British Journal of Cancer (1998) 77(11), 2045–2047
© 1998 Cancer Research Campaign

### No association between a polymorphism in the steroid metabolism gene *CYP17* and risk of breast cancer

**AM Dunning[1*], CS Healey[1*], PDP Pharoah[1,3*], NA Foster[1], JM Lipscombe[3], KL Redman[2], DF Easton[2], NE Day[3] and BAJ Ponder[1]**

[1]CRC Human Cancer Genetics Research Group, Box 238, Addenbrooke's Hospital, Cambridge CB2 2QQ; [2]CRC Genetic Epidemiology Unit and [3]University of Cambridge Department of Community Medicine, Strangeways Research Laboratories, Worts Causeway, Cambridge CB1 4RN, UK

**Summary** A recent study showed an association between a single base substitution, T→C, in the promotor region of the *CYP17* gene, the risk of breast cancer and age at menarche in Asian, African–American and Latino women from California and Hawaii. The C allele was associated with increased risk of breast cancer, significantly so for patients presenting with advanced disease, whereas the TT genotype was associated with later age at menarche in control subjects. We attempted to confirm these findings in a large case–control study in East Anglia, England (835 cases and 591 control subjects). We found no evidence of an increased risk of breast cancer [odds ratio (OR) 1.10, confidence interval (CI) 0.89–1.37] or advanced breast cancer (OR 0.88, CI 0.38–2.01) in C allele carriers, nor any association between age at menarche and genotype. We conclude that these alleles do not significantly alter breast cancer risk in the English population.

**Keywords:** 617 gene polymorphism; steroid metabolism; breast cancer

Epidemiological and laboratory studies indicate that breast cancer risk is strongly related to hormonal factors, specifically exposure to endogenous oestrogens. Ecological studies indicate that hormone levels are strongly influenced by lifestyle factors (e.g. diet), but genetic factors could also be important. This raises the possibility that polymorphisms in genes involved in sex steroid hormone metabolism may have a role in breast cancer susceptibility. One such gene is *CYP17*, which encodes an enzyme, cytochrome P450c17α. This enzyme has two different roles in steroid interconversion: the 17α hydroxylase activity can convert progesterone to 17α-hydroxyprogesterone, and the 17,20 lyase function may further convert 17α-hydroxyprogesterone to androstenedione (the precursor of both oestrone and testosterone). There is a polymorphic T to C substitution 34 bp upstream of the translation initiation site in the promotor region of the gene, which creates an MspA1 restriction site (Carey et al, 1997). This substitution also creates a fifth Sp1-type (CCACC) motif in the 5′ region of the *CYP17* gene. It is thought that gene transcription may correlate with the number of these motifs, and so this polymorphism has the potential to alter the promotor activity and possibly also the production rate of *CYP17*.

Two association studies using this *CYP17* MspA1 restriction fragment length polymorphism (RFLP) have been published to date. One study investigated the association between genotype and polycystic ovary and male pattern baldness (Carey et al, 1997). Carriers of the C allele were found to have a twofold increased risk of either polycystic ovary disease or male pattern baldness, depending on sex. More recently, Feigelson et al (1997) reported an association of the same C (A2) allele with an increased risk of breast cancer, which was significant only in patients presenting with advanced breast cancer. They also found that within their control group the TT (A1) homozygotes had a later age at menarche than carriers of the C allele and they suggested that the reduced risk of breast cancer associated with later age at menarche is limited to TT homozygous women. However, this study was performed using only 174 breast cancer cases and 285 control subjects from three different ethnic groups and significant results were only demonstrated in subgroup analyses. We set out to confirm these findings in a much larger case–control study from the East Anglian region of England.

*Received 27 November 1997*
*Revised 1 December 1997*
*Accepted 9 December 1997*

*Correspondence to:* AM Dunning

### MATERIALS AND METHODS

### Case and control subject selection

Cases were taken from a population-based study of breast cancer. All women in the region served by the Anglian cancer registry with breast cancer diagnosed between 1 January 1991 and 30 June 1996 who were under the age of 55 at diagnosis were eligible to take part. Genotyping was carried out on the first 864 samples received from 2007 eligible patients. Control subjects were randomly selected from the UK part of the European Prospective Investigation of Cancer (EPIC) (Riboli and Kaaks, 1997), a prospective study of diet and cancer being carried out in the same population from which the cases have been drawn. The EPIC cohort comprises 25 000 individuals resident in Norfolk (East Anglia), aged 45–74 years.

### Genotype detection

The *CYP17*, 5′-utr, MspA1 polymorphism assay has been described previously (Carey et al, 1997; Feigelson et al, 1997). Briefly, a 459-bp polymerase chain reaction (PCR) product was amplified using the following primer pair: *CYP17*-F, 5′-CATTCG-CACCTCTGGAGTC and *CYP17*-R, 5′-GGCTCTTGGGGTAC-TTG, and the following conditions on a TouchDown thermal

*Contributed equally to this study.

Table 1 Genotype frequencies and associated breast cancer risks

| Genotype | Controls (*n*) | Cases (*n*) | Odds ratio | 95% CI |
|---|---|---|---|---|
| TT | 229 | 303 | 1.00 | 0.81–1.24[a] |
| TC | 277 | 402 | 1.09 | 0.86–1.36[a] |
| CC | 85 | 130 | 1.17 | 0.92–1.49[a] |
| TC/CC | 362 | 532 | 1.10 | 0.89–1.37 |

[a]Floated confidence interval (see text).

Table 2 Risk of breast cancer in women with late age at menarche (≥ 13 years) by genotype

| Genotype | Adjusted OR[a] | 95% CI |
|---|---|---|
| All | 0.94 | 0.71–1.26 |
| TT | 0.93 | 0.57–1.51 |
| TC | 0.95 | 0.61–1.46 |
| CC | 0.59 | 0.27–1.27 |

[a]Adjusted for age.

cycler (Hybaid, UK) with the use of the hot lid: 95°C, 20 min, one cycle; 94°C, 1 min, 57°C, 1 min, 72°C, 1 min, 35 cycles; 72°C, 10 min, one cycle. The reaction was carried out in a 50-µl volume, using 50 ng of genomic DNA template, 200 µM dNTP, 3 mM $MgCl_2$, 200 nM of each primer, 0.6 U of Amplitaq Gold (Perkin Elmer, UK) and 1 × Amplitaq Gold buffer. MspA1 (NEB) digests were carried out in a 20-µl volume according to manufacturer's instructions and separated on a 2% agarose gel (Gibco BRL).

### Statistical analysis

The association between *CYP17* alleles and breast cancer was assessed using $\chi^2$ tests. For the three-way comparison between genotype and risk of breast cancer, variances were estimated by treating odds ratios as floating absolute risks (Easton et al, 1991). This approach yields floated standard errors and floated confidence intervals. Although the method does not alter the relative risk estimates, it reduces the variances attributed to the odds ratios that are not defined as 1.00, and also reduces unwanted covariance between them. This allows a valid comparison between the two non-baseline groups. A logistic regression model was used to assess a potential interaction between genotype, age at menarche and the risk of breast cancer. In this analysis, age was adjusted in 5-year strata.

### RESULTS

The results of genotyping our case–control series for the T to C polymorphism are shown in Table 1. The genotype distribution in control subjects was very close to that expected under Hardy–Weinberg equilibrium. We detected no significant differences in either the allele frequencies ($\chi^2 = 0.85$, 1 d.f., $P = 0.35$) or the genotype distributions between breast cancer cases and the control subjects ($\chi^2 = 0.86$, 2 d.f., $P = 0.65$). Table 1 also shows the genotype-specific relative risks of breast cancer as estimated by the odds ratio (OR). No significant effect of genotype on age was found in either control subjects or cases, and so unadjusted odds ratios are presented. Cases were also divided into three subgroups according to stage at diagnosis: stage I ($n = 367$), stage II ($n = 411$) and stage III/IV ($n = 24$). Again no significant effects were found, with the relative risks of the three types of breast cancer in women carrying the C allele being 0.95 (0.73–1.24), 1.23 (0.98–1.54) and 0.88 (0.38–2.01) respectively.

No effect of genotype on age at menarche was found in the control population: the mean ages at menarche were 12.94 years (12.74–13.14) in TT homozygous women, 12.96 (12.74–13.18) in heterozygotes and 12.95 (12.56–13.34) in CC homozygotes (one-way ANOVA, $P = 0.77$). In addition, there was no significant association between genotype and age at first full-term pregnancy or parity.

The relative risks of breast cancer associated with late age at menarche (≥ 13 years) stratified by genotype are shown in Table 2. We found a non-significant reduction in breast cancer risk in individuals with late age at menarche. There was no difference in this risk when stratified by genotype, a finding consistent with the lack of association between genotype and age at menarche in control subjects.

### DISCUSSION

We found no significant effect of the *CYP17* promotor T to C substitution on breast cancer risk in a large case–control study, nor have we been able to confirm the previously reported association between the *CYP17* genotype and risk of advanced breast cancer: Feigelson et al (1997) reported a significant association of the C allele with advanced disease (OR 2.5, 95% CI 1.07–5.94), indicating that disease progression may be more rapid in carriers of the C allele. In contrast, we found a reduced risk of advanced disease in C allele carriers. However, the number of cases with advanced disease in each study was small, the associated confidence intervals are wide and there was no significant difference between the two risk estimates ($\chi^2 = 3.00$, $P = 0.082$). Combining the data from the two studies gives an OR for advanced cancer in women carrying the C allele of 1.57 (0.89–2.90), and so the data are compatible with a small increase in risk of advanced cancer. A larger study addressing the issue of cancer progression would be required to confirm this.

Late age at menarche has been shown to be associated with a reduced risk of breast cancer (Kelsey et al, 1993), and the results of this study are consistent with this effect. Unlike Feigelson et al (1997) we found no effect of genotype on age at menarche. In our control subjects, mean age at menarche was 0.02 years (95% CI – 0.30 to 0.27) earlier in TT homozygotes than in women carrying a C (A2) allele, whereas Feigelson et al reported that the mean age at menarche in TT (A1A1) women was 0.4 years later. From our result we would not expect to find an effect of genotype on risk of breast cancer associated with late age at menarche, and no such effect was demonstrated.

Our study had an 80% power to detect a 1.3-fold increased risk in C-allele carriers at a significance level of 5%, which is the magnitude of the effect reported in the study by Feigelson et al (1997). However, we failed to confirm their reported association between *CYP17* genotype, age at menarche and breast cancer risk. There are several possible explanations for the difference between our results and those of Feigelson et al. The ethnic background of the two study populations are different (the population of East Anglia is close to 100% white), and, although the *CYP17* genotype distribution in East Anglia is similar to that of the American control subjects, there is the potential for as yet unidentified gene–gene interactions between *CYP17* and genes that differ in

© Cancer Research Campaign 1998

frequency in the two populations. Alternatively, it is possible that the T→C substitution is not in itself disease causing, but in some populations is in linkage disequilibrium with another alteration that is disease causing. If this were true, then the association would only be apparent in those populations. Another possible explanation is that genotype effects are age specific, with a marked effect only in older cases. Further studies of older cases would be needed to address this possibility. The most likely explanation is that the original study was small and the positive associations reported were chance findings that have not been confirmed by a larger and more powerful study.

### ACKNOWLEDGEMENTS

We thank Robert Lubin and Suzy Oakes for access to EPIC samples and data, and Patricia Harrington, Simon McBride and Paul Russell for help in preparing DNA samples. The CRC Human Cancer Genetics Group and the CRC Genetic Epidemiology Unit are supported by grants from the Cancer Research Campaign, case collection is supported by a grant from the NHS Research and Development and EPIC is supported by grants from the Medical Research Council, the Cancer Research Campaign and the European Union. BAJP is a Gibb Fellow of the CRC.

### REFERENCES

Carey AH, Waterworth D, Patel K, White D, Little J, Novelli P, Franks S and Williamson R (1997) Polycystic ovaries and premature male-pattern baldness are associated with one allele of the steroid metabolism gene CYP17. *Hum Mol Genet* **10**: 1873–1876

Easton DF, Peto J and Babiker AGAG (1991) Floating absolute risk: an alternative to relative risk in survival and case–control analysis avoiding an arbitrary reference group. *Stat Med* **10**: 1025–1035

Feigelson HS, Coetzee GA, Kolonel LN, Ross RK and Henderson BE (1997) A polymorphism in the CYP17 gene increases the risk of breast cancer. *Cancer Res* **57**: 1063–1065

Kelsey JL, Gammon MD and John EM (1993) Reproductive factors and breast cancer. *Epidemiol Rev* **15**: 36–47

Riboli E and Kaaks R (1997) The EPIC project: rationale and study design. *Int J Epidemiol* **26**: S6–S14

© Cancer Research Campaign 1998